# Structural equation model of factors related to quality of life for community-dwelling schizophrenic patients in Japan

**DOI:** 10.1186/1752-4458-8-32

**Published:** 2014-07-25

**Authors:** Hirofumi Nakamura, Naoko Watanabe, Eisuke Matsushima

**Affiliations:** 1 Section of Liaison Psychiatry and Palliative Medicine, Graduate School of Medical and Dental Sciences, Tokyo Medical and Dental University, 1-5-45 Yushima, Bunkyo-ku, Tokyo 113-8519, Japan; 2 Faculty of Nursing, Department of Nursing, Josai International University, 283-8555 1 Gumyo, Togane-City, Chiba, Japan; 3 Ibaraki Prefectural University of Health Sciences, 4669-2 Ami, Ami-machi, 300-0394 Inashiki-gun, Ibaraki, Japan

**Keywords:** Quality of life, Schizophrenia, Self-efficacy, Self-esteem, Regression analysis, Structured equation modeling

## Abstract

**Background:**

This study aimed to clarify how community mental healthcare systems can be improved.

**Methods:**

We included 79 schizophrenic patients, aged 20 to 80 years, residing in the Tokyo metropolitan area who regularly visited rehabilitation facilities offering assistance to psychiatric patients and were receiving treatment on an outpatient basis. No subjects had severe cognitive disorders or were taking medication with side effects that could prevent the completion of questionnaires. Questionnaires included items related to quality of life, self-efficacy, self-esteem, psychosis based on the Behavior and Symptom Identification Scale, health locus of control, and socio-demographic factors. We performed multiple linear regression analysis with quality of life as the dependent variable and, based on covariance structural analysis, evaluated the goodness of fit of the resulting structural equations models.

**Results:**

Self-efficacy, self-esteem, and degree of psychosis significantly impacted quality of life. Marital status, age, and types of medications also influenced quality of life. Multiple linear regression analysis revealed psychiatric symptoms (Behavior and Symptom Identification Scale-32 [daily living and role functioning] (Beta = −0.537, p < 0.001) and self-efficacy (Beta = 0.249, p < 0.05) to be predictors of total quality of life score. Based on covariance structural analysis, the resulting model was found to exhibit reasonable goodness of fit.

**Conclusions:**

Self-efficacy had an especially strong and direct impact on QOL. Therefore, it is important to provide more positive feedback to patients, provide social skills training based on cognitive behavioral therapy, and engage patients in role playing to improve self-efficacy and self-concept.

## Background

Efforts are being made in the area of mental health care in Japan to shorten the length of hospital stays and encourage discharge of patients hospitalized over the long term. These steps increase the importance of establishing community-based facilities to care for and provide support to psychiatric patients. Efforts must not only provide support for the transition to life in communities but must extend to providing support for maintaining life in and improving quality of life (QOL) of psychiatric patients in communities. To this aim, it is necessary to understand schizophrenic patients’ perceptions and feelings regarding QOL and develop specific means of support. Given that multiple factors impact the QOL of schizophrenic patients, evaluation of QOL must not be limited to a single dimension such as symptom stability, but should include subjective as well as social factors.

Previous studies have assessed factors impacting the QOL of schizophrenic patients. Gait et al. [1] investigated the QOL of schizophrenic patients in 5 European countries and found that psychiatric symptoms, frequency of contact with friends and family, and age were significantly related to subjective QOL. Level of satisfaction with support services and sex were also found to significantly influence subjective QOL in some countries. Focusing on depressive and negative symptoms, Fitzgerald et al. [2] compared patient-rated QOL assessed by schizophrenic patients themselves and QOL evaluated by care providers. They found that subjective QOL was associated with depressive symptoms while observer-rated QOL was associated with both depressive and negative symptoms. Rinsner [3] investigated subjective QOL of schizophrenic patients at admission and 16 months after discharge and found that satisfaction with leisure activities and support services was related to improved QOL, and that the impact of these factors was greater than that of psychiatric symptoms. Huppert et al. [4] studied 63 schizophrenic patients being treated on an outpatient basis and found that depression was significantly associated with subjective QOL, and that no schizophrenic symptoms were associated with subjective QOL.

In Japan, Isoishi et al. [5] studied the QOL of schizophrenic patients receiving day care services and reported that, while psychological factors and negative symptoms, low self-esteem, and the existence of a place to stay substantially impacted subjective QOL, it was not affected by impairments in daily life. Suzuki et al. [6] proposed “perceived difficulty” as an indicator of subjective QOL and developed a questionnaire with 5 levels of questions in 6 categories. They found that degree of perceived difficulty was significantly correlated with observer-rated QOL as well as severity of depression and anxiety. They also reported that among the factors examined, changes in negative symptoms were correlated with changes in perceived difficulty. Meanwhile, they found that the correlation coefficient between the empowerment scale score and severity of psychiatric symptoms was small and concluded that the empowerment score was, for the most part, independent from psychiatric symptoms. No Japanese researcher has hypothesized a predictor of subjective QOL other than psychiatric symptoms and socio-demographic background. Identifying factors that contribute to QOL of community-dwelling schizophrenic patients and understanding the structural relationships of these factors is necessary to improve the quality of community-based mental health services.

This study aimed to multi-dimensionally and structurally analyze factors related to QOL of community-dwelling schizophrenic patients and gain insight into how community-based mental health care can be improved.

## Methods

### Participants

Subjects included 79 schizophrenic patients living in the Tokyo metropolitan area who were not currently hospitalized and who regularly visited rehabilitation facilities offering assistance to psychiatric patients (community living support centers, group homes for psychiatric patients, and facilities providing support for vocational transition and continuation of employment, etc.). Patients were between 20 and 80 years of age, had been diagnosed with schizophrenia, were receiving treatment on an outpatient basis, were determined to have the mental and physical capacity to participate in the study, did not suffer from severe cognitive disorders, and were not taking medication with severe enough side effects to prevent the filling out of questionnaires. The investigation was conducted between April 2010 and October 2011.

### Procedure

Study subjects were recruited by directors and persons in charge of various rehabilitation facilities. These individuals identified patients meeting requirements for inclusion in the study and believed to be appropriate subjects. After receiving a verbal explanation of the purpose of the study, patients agreeing to participate were introduced to the researchers. The objective of the study was then explained by researchers in detail using written materials, and patients then signed a written agreement to participate in the study.

### Measurements

#### Quality of Life

To assess QOL, we used a life satisfaction measure for psychiatric patients that consisted of 31 items in 6 categories, including 1 item related to life in general, 5 items related to health and physical function, 7 items related to living environment, 6 items related to social living skills, 4 items related to interactions with others, and 8 items related to psychological function. Responses were scored on a 7-point Likert scale ranging from “extremely dissatisfied” (−3), “dissatisfied” (−2), “somewhat dissatisfied” (−1), “neither dissatisfied nor satisfied” (0), “basically satisfied” (+1), “satisfied” (+2), and “extremely satisfied” (+3). This is a reliable and validated measure to assess the QOL of psychiatric patients [7]. Reliability of the measure was assessed by calculating Cronbach’s alpha to check for internal consistency. Crobach’s alpha coefficient for the subscales was 0.73–0.82. Validity of the measure has previously been demonstrated based on significant correlation with Chicago University’s Life Satisfaction Index with the Global Assessment of Functioning.

#### Self-perception

We used Rosenberg’s Self Esteem Scale [8] to assess patients’ self-esteem. Self-esteem encompasses various elements, including self-respect and proprioception, and refers to individuals’ feelings about themselves as well as their senses and feelings about their worth and ability. Rosenberg posits that self-esteem does not refer to the sense of superiority or inferiority that comes from comparing oneself to others but, rather, the degree of self-respect and self-evaluation of one’s worth. The term “self-esteem” has two meanings, depending on context, either of having an “extremely high sense of self-worth” or a sense that “this is okay”. It is this latter sense that indicates degree of self-esteem. The scale does not include subscales and consists of 10 items scored on a 5-point Likert scale with responses ranging from “agree” to “disagree”. A higher score represents a higher sense of self-esteem. Reliability and validity of this measure have previously been established. Crobach’s alpha coefficient was 0.72.

We used the General Self Efficacy Scale (GESE) scale developed by Sakano et al. [9] to assess patients’ self-efficacy. Self-efficacy refers to the confidence that individuals have in themselves that they can carry out actions necessary to achieve a certain result [10]. In addition, self-efficacy can be used to rationally explain, evaluate, and regulate processes involving a wide range of changes in behavior. The measure consists of 16 items scored on a 2-point Likert scale, including 7 items related to “aggressiveness of action,” 5 items related to “anxiety regarding failure,” and 3 subscales of 4 items related to “social status in terms of individual skills and ability”. Reliability and validity of the scale was established by Sakano et al. [9]. In this scale, lower scores indicate greater anxiety; however, the “anxiety regarding failure” subscale in the GESE is reverse-scored, meaning that higher scores indicate lower anxiety. Crobach's alpha coefficient for the subscales in this study was 0.68–0.77.

#### Health locus of control

We used the Japanese version of the Health Locus of Control Scale [11] to evaluate health locus of control. Rotter [12] focused on individuals’ behavior based on their beliefs regarding causal relationships as well as their beliefs regarding their ability to control the intensity of such relationships. Wallston et al. [13] applied this theory to develop a scale to measure beliefs regarding the causes of illness and health. They also explained that, in the context of medical care, understanding what kind of beliefs a patient has regarding health is important for implementing an individualized approach to medical treatment. Horie [11] developed a Japanese version of this scale with modified questions easily comprehensible in Japanese. The scale consists of 25 items scored on a 6-point Likert scale constituting 5 subscales related to self (internal), family, profession, chance, and the supernatural. This scale indicated a greater tendency to attribute health to internal factors. A higher score represents a positive way of thinking regarding the subscales. Reliability and validity of the scale have previously been established. Crobach’s alpha coefficient for the subscales was 0.56–0.77.

#### Behavior and symptoms

We used the Behavior and Symptom Identification Scale (BASIS-32) [14] to evaluate psychiatric symptoms. The BASIS-32 was devised to evaluate the outcomes of psychiatric treatment as perceived by patients. Briefly, it is designed to comprehensively measure major psychoses and degree of difficulty in various functional domains based on self-reports by patients. The scale, developed by Eisen, consists of 32 items scored on a 5-point Likert scale, with scores ranging from 0 to 4.

Unique features of BASIS-32 include the following: (1) it is based on patients’ perceptions, (2) it comprises 5 subscales of multidimensional psychiatric symptoms, (3) it is a comprehensive enough for use with various psychiatric diseases, (4) it was developed as an index of medical outcomes, and (5) it is simple and easy to complete.

Reliability and validity of the scale have previously been established. Crobach’s alpha coefficient for the subscales was 0.89–0.93.

BASIS-32 is a multidimensional measure of behavior and symptoms designed to evaluate the psychiatric symptoms of patients in 5 domains: (1) relation to self and others, (2) daily living skills, (3) depression and anxiety, (4) impulsive and addictive behavior, and (5) psychosis. The score for each subscale represents the mean score of items from which the subscale is constructed. For any given domain, higher scores indicate more severe symptoms and perceived problems/difficulties.

#### Patients’ medical histories and demographic factors

The community-dwelling schizophrenic patients were also asked questions about their medical histories, including the name of their illness, age at onset, number of hospitalizations, current status of symptoms, medications taken, side effects of medications taken, tobacco use, and alcohol use. In addition, patients were asked questions related to demographic factors including age, sex, living situation, marital status, educational level, and type of residence.

### Statistical analyses

We performed appropriate statistical analyses to cross-sectionally examine the QOL, characteristics of QOL, and the medical histories of community-dwelling schizophrenic patients. After generating descriptive statistics, we first analyzed differences in responses based on descriptive data. Subsequently, after confirming a normal distribution of scores for each measure, we examined the relationship between independent (patients’ medical histories and characteristics) and dependent (patients’ QOL) variables based on correlation and multiple linear regression analyses. Multiple linear regression analysis was necessary to elucidate the factors by which the dependent variables might potentially be influenced, and to what extent. These findings were then used as the basis for covariance structure analysis. Based on these analyses, we specified a structural equation model (SEM) and, after modifying the model to optimize fit, clarified the mutual relationship between factors. Goodness of fit of the model was evaluated based on the χ^2^, goodness of fit index (GFI), adjusted goodness of fit index (AGFI), comparative fit index (CFI), and root mean square error of approximation (RMSEA). Satisfactory goodness of fit was defined as χ^2^/df < 2.0, GFI > 0.95, AGFI > 0.90, CFI > 0.97, and RMSEA < 0.05, and acceptable goodness of fit was defined as χ^2^/df < 3.0, GFI > 0.90, AGFI > 0.85, CFI > 0.95, and RMSEA < 0.08. We further improved the model by removing non-significant paths and comparing the fit of multiple models using the Akaike Information Criteria [15]. Power analysis was performed to test the sample size. With a median effect size and a significance level of 5% in a two-tailed test, following Cohen’s criteria for effect size, the total sample size required was ≥55 for a power of 0.8, confirming the validity of this study’s sample size of 79.

### Ethical considerations

This study was approved by the Tokyo Medical and Dental University Faculty of Medicine Ethics Committee (approval number 810). The study sample consisted of patients from whom written agreement to participate in the study was obtained after the patients received an explanation, at the time of recruitment, that analyses would be performed in such a manner that individuals could not be identified, that participants could, at any time, freely withdraw from the study, that participation or non-participation in the study would not negatively impact their relation with rehabilitation centers, and that the results of the study would be presented at conferences and through other venues.

### Consent

Written informed consent was obtained from the patient for the publication of this report and any accompanying images.

## Results

### Characterization of the sample

Baseline characteristics of study participants are shown in Table 1. Mean (SD) age of participants was 42.6 ± 13.0 years. Mean age-of-onset was 23.4 ± 10.2 years; mean number of hospitalizations was 2.3 ± 3.2, mean types of medications taken was 4.9 ± 4.3, and mean number of cigarettes smoked per day was 21.6 ± 12.9. About half of the participants stated that they experienced side effects of medication. The population also included a somewhat high percentage of smokers (40%). In terms of drinking, 60% of participants answered that they did not drink alcohol. Household size was 2 or more people for 60% of participants. In terms of marital status, an overwhelming majority (>90%) were single. However, this is the average figure for people with mental health problems in Japan.

**Table 1:** Baseline characteristics of study participants

**n = 79**					
	**n**	**%**		**n**	**%**
**Age, yr**			**Side effects from medications**		
20-29	9	11.4	Yes	43	54.4
30-39	27	34.2	No	35	44.3
40-49	17	21.5	No response	1	1.3
50-59	15	18.9			
60-69	10	12.7	**Tobacco use**		
70-79	1	1.3	Yes	32	40.5
			No	47	59.5
**Sex**					
Male	45	57.0	**Alcohol use**		
Female	34	43.0	Daily	1	1.3
			Occasionally	28	35.4
**Age at disease onset, yr**			None	50	63.3
≤15	7	8.9			
16-20	21	26.6	**Living situation**		
21-25	23	29.1	Live alone	27	34.2
26-30	10	12.7	Live with two or more persons (including family)	50	63.3
31-40	11	13.9	Live in a facility	2	2.5
≥41	5	6.3			
No response	2	2.5	**Marital status**		
			Married	6	7.6
**Number of hospitalizations**			Single	73	92.4
0	12	15.2			
1	25	31.6	**Level of education**		
2	10	12.7	Graduated junior high school	12	15.2
3	9	11.4	Graduated high school	45	57.0
4	4	5.1	Graduated university	21	26.5
≥5	13	16.4	No response	1	1.3
No response	6	7.6			
			**Residence type**		
**Types of medicines taken internally**			Single-family home	42	53.2
1	11	13.9	Apartment building	35	44.3
2	5	6.3	Facility	2	2.5
3	11	13.9			
4	15	19.0			
≥5	31	39.3			
No response	6	7.6			

### Multiple linear regression of QOL

Multiple linear regression analysis using schizophrenic patients’ QOL as the dependent variable and patients’ scores on other measures as independent variables revealed that QOL (life in general) was significantly influenced by self-esteem (β = 0.463, p < 0.01), age of onset (β = −0.333,p < 0.01), and self-efficacy (social position and ability) (β = −0.285, p < 0.05), types of medications taken (β = −0.278, p < 0.05), and health locus of control (HLC) (family) (β = 0.236, p < 0.05). Similarly, QOL (physical functioning) was influenced by BASIS (daily living and role functioning) (β = −0.557, p < 0.001); QOL (environment) was influenced by BASIS (daily living and role functioning) (β = −0.611, p < 0.001); QOL (social skills) was influenced by BASIS (daily living and role functioning) (β = −0.494, p < 0.001), self-efficacy (Total) (β = 0.275, p < 0.01), and HLC (internal)] (β = 0.179, p < 0.05); QOL (social relationships) was influenced by BASIS (relation to self and others) (β = −0.556, p < 0.01) and self-efficacy (social position and ability) (β = 0.309, p < 0.01); QOL (psychological functioning) was influenced by BASIS (daily living and role functioning) (β = −0.489, p < 0.001), self-efficacy (total) (β = 0.321, p < 0.01), and age of onset (β = −0.190, p < 0.05); and QOL (total score) was influenced by BASIS (daily living and role functioning) (β = −0.537, p < 0.001) and self-efficacy (β = 0.249, p < 0.05) (Table 2).

**Table 2:** Stepwise multiple regression analysis for quality of life (QOL)

			**N = 66**
**QOL dependent variable**	**Independent variable**	**Adjusted R**^**2**^	**β**
Life in general	Self-esteem	R^2^ = 0.288***	0.463**
	Age at onset		−0.333**
	Self-efficacy (social position and ability)		−0.285*
	Type of medications taken		−0.278*
	Health Locus of Control (family)		0.236*
Physical functioning	BASIS-32 (daily living and role functioning)	R^2^ = 0.300***	−0.557***
Environment	BASIS-32 (daily living and role functioning)	R^2^ = 0.364***	−0.611***
Social skills	BASIS-32 (daily living and role functioning)	R^2^ = 0.551***	−0.494***
	Self-efficacy total		0.275**
	Health Locus of Control (internal)		0.179*
Social relationships	BASIS-32 (relationship to self and others)	R^2^ = 0.430***	−0.556***
	Self-efficacy (social position and ability)		0.309**
Psychological functioning	BASIS-32 (daily living and role functioning)	R^2^ = 0.459***	−0.489***
	Self-efficacy total		0.321**
	Age at onset		−0.190*
Total score	BASIS-32 (daily living and role functioning)	R^2^ = 0.476***	−0.537***
	Self-efficacy total		0.249*

BASIS, Behavior and Symptom Identification Scale; QOL, Quality of Life; R2, coefficient of determination.

β, standard partial regression coefficient.

*p < 0.05 **p < 0.01 ***p < 0.001.

### Multiple linear regression of BASIS-32

Multiple linear regression analysis using schizophrenic patients’ BASIS (behavior and symptoms) as the dependent variable and patients’ scores on other measures as independent variables revealed that BASIS (relation to self and others) was significantly influenced by QOL (total score) (β = −0.612, p < 0.001), self-esteem (β = −0.412, p < 0.01), self-efficacy (social position and ability) (β = 0.310, p < 0.01), and QOL (life in general) (β = −0.247, p < 0.05). Similarly, BASIS (daily living and role functioning) was influenced by QOL (social skills) (β = −0.499, p < 0.001) and self-esteem (β = −0.336, p < 0.01); while BASIS (depression and anxiety) was influenced by QOL (total score) (β = −0.471, p < 0.001) and self-esteem (β = −0.303, p < 0.01); BASIS (impulsive and addictive behavior) was influenced by QOL (social skills) (β = −0.521, p < 0.001); and BASIS (psychosis) was influenced by self-esteem (β = −0.419, p < 0.001), QOL (environment) (β = −0.348, p < 0.01), and self-efficacy (social position and ability) (β = 0.243, p < 0.05) (Table 3).

**Table 3:** Stepwise multiple regression analysis for BASIS-32

			**N = 66**
**BASIS-32 dependent variable**	**Independent variable**	**Adjusted R**^**2**^	**β**
Relationship to self and others	QOL total score	R^2^ = 0.474***	−0.612***
	Self-esteem		−0.412**
	Self-efficacy (social position and ability)		0.310**
	QOL (life in general)		0.247*
Daily living and role functioning	QOL (social skills)	R^2^ = 0.529***	−0.499***
	Self-esteem		−0.336**
Depression and anxiery	QOL total score	R^2^ = 0.460***	−0.471***
	Self-esteem		−0.303**
Impulsive and addictive behavior	QOL (social skills)	R^2^ = 0.261***	−0.521***
Psychosis	Self-esteem	R^2^ = 0.318***	−0.419**
	QOL (environment)		−0.348**
	Self-efficacy (social position and ability)		0.243*

BASIS, Behavior and Symptom Identification Scale; QOL, Quality of Life; R2, coefficient of determination.

β, standard partial regression coefficient.

*p < .05 **p < .01 ***p < .001.

### Multiple linear regression of self-efficacy

Multiple linear regression analysis using schizophrenic patients’ self-efficacy as the dependent variable and patients’ scores on other measures as independent variables revealed that self-efficacy (aggressiveness of action) was significantly influenced by self-esteem (β = 0.444, p < 0.001), QOL (social skills) (β = 0.310, p < 0.01), HLC (internal) (β = 0.266, p < 0.01), and QOL (environment) (β = −0.216, p < 0.01). Similarly, self-efficacy (anxiety related to failure) was found to be influenced by self-esteem (β = 0.602, p < 0.001) and age (β = 0.195, p < 0.05); self-efficacy (social position and ability) was influenced by self-esteem (β = 0.629, p < 0.001), QOL (social skills) (β = 0.431, p < 0.001), BASIS (relation to self and others) (β = −0.330, p < 0.01, QOL (life in general) (β = −0.316, p < 0.01), and age of onset (β = −0.293, p < 0.01); self-efficacy (total score) was influenced by self-esteem (β = 0.550, p < 0.001), QOL (social skills) (β = 0.490, p < 0.01), and QOL (environment) (β = −0.256, p < 0.05) (Table 4). It should be noted that here, the “anxiety related to failure” subscale in the GESE is reverse-scored, meaning that higher scores indicate lower anxiety (Table 4).

**Table 4:** Stepwise multiple regression analysis for self-efficacy

			**N = 66**
**Self-efficacy dependent variable**	**Independent variable**	**Adjusted R**^**2**^	**β**
Aggressiveness of action	Self-esteem	R^2^ = 0.510***	0.444***
	QOL (social skills)		0.310**
	Health Locus of Control (internal)		0.266**
	QOL (life in general)		−0.216*
Anxiety to failure	Self-esteem	R^2^ = 0.408***	0.602***
	Age		0.195*
Social position of ability	Self-esteem	R^2^ = 0.476***	0.629***
	QOL (social skills)		0.431**
	BASIS-32 (relationship to self and others)		0.330**
	QOL (life in general)		−0.316**
	Age at onset		−0.293**
Total	Self-esteem	R^2^ = 0.579***	0.550***
	QOL (social skills)		0.490***
	QOL (environment)		−0.256*

BASIS, Behavior and Symptom Identification Scale; QOL, Quality of Life; R2, coefficient of determination.

β, standard partial regression coefficient.

*p < .05**p < .01 ***p < .001.

### Multiple linear regression of self-esteem

Multiple linear regression analysis using schizophrenic patients’ self-esteem as the dependent variable and patients’ scores on other measures as independent variables revealed that self-esteem was significantly influenced by self-efficacy (total score) (β = 0.537, p < 0.001) and BASIS (daily living and role functioning) (β = −0.321, p < 0.01) (Table 5).

**Table 5:** Stepwise multiple regression analysis for self-esteem

			**N = 66**
**Dependent variable**	**Independent variable**	**Adjusted R**^**2**^	**β**
Self-esteem	Self-efficacy total	R^2^ = 0.561***	0.537***
	BASIS-32 (daily living and role functioning)		−0.321**

BASIS, Behavior and Symptom Identification Scale; R2, coefficient of determination;

β, standard partial regression coefficient.

**p < .01 ***p < .001.

### Multiple linear regression of health locus of control

Multiple linear regression analysis using schizophrenic patients’ HLC as the dependent variable and patients’ scores on other measures as independent variables revealed that HLC (internal) was significantly influenced by self-efficacy (aggressiveness of action) (β = 0.425, p < 0.001). Similarly, HLC (family) was found to be influenced by QOL (environment) (β = 0.275, p < 0.05), while HLC (professional) was influenced by QOL (environment) (β = 0.807, p < 0.001), QOL (total score) (β = −0.605, p < 0.05), and number of hospitalizations (β = 0.301, p < 0.05) (Table 6).

**Table 6:** Stepwise multiple regression analysis for Health Locus of Control (HLC)

			**N = 66**
**HLC dependent variable**	**Independent variable**	**Adjusted R**^**2**^	**β**
Internal	Self-efficacy (aggressiveness of action)	R^2^ = 0.168***	0.425***
Family	QOL (environment)	R^2^ = 0.061*	0.275*
Professional	QOL (environment)	R^2^ = 0.176**	0.807***
	QOL total score		−0.605*
	Number of hospitalizations		0.301*

QOL, quality of life; R2, coefficient of determination.

β, standard partial regression coefficient.

*p < .05 **p < .01 ***p < .001.

### Factors determining QOL of psychiatric patients identified through covariance structural analysis

**Figure 1:**
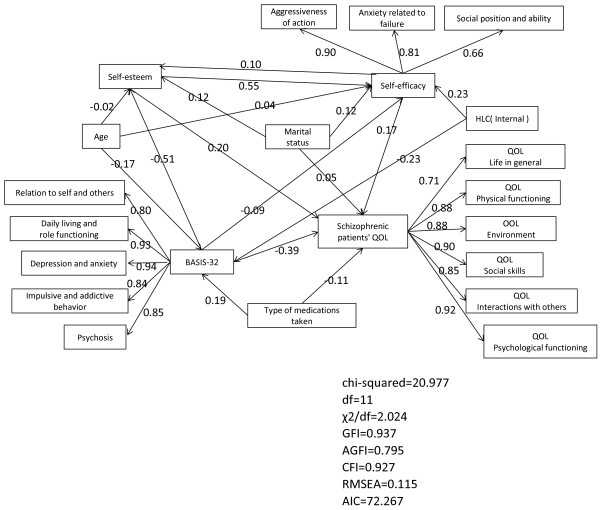
**Initial model (Structural equation model).** Measures of model fit (χ^2^/df = 2.024, GFI = 0.937, AGFI = 0.795, CFI = 0.927, RMSEA = 0.115) indicated low goodness of fit of the initial model. The AIC for the initial model was 72.267.

Based on the above results, in order to examine the causal relationship among factors determining QOL, we developed an SEM and performed covariance structural analysis. In the initial model, we assumed that self-efficacy affects self-esteem, that psychiatric symptoms (BASIS-32) affect self-efficacy, and that these factors indirectly influence QOL. In terms of socio-demographic factors, we assumed that patient’s age influences self-esteem and self-efficacy and that marital status affects QOL. Measures of model fit (χ^2^/df = 2.024, GFI = 0.937, AGFI = 0.795, CFI = 0.927, RMSEA = 0.115) indicated low goodness of fit of the initial model. The AIC for the initial model was 72.267 (Figure 1).

**Figure 2:**
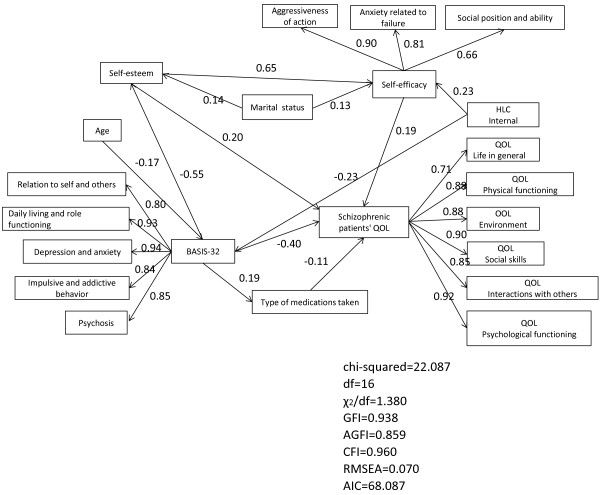
**Revised model (Structural equation model).** Measures of model fit (χ^2^/df = 1.380, GFI = 0.938, AGFI = 0.859, CFI = 0.960, RMSEA = 0.070) indicated that the revised model had a satisfactory fit. The AIC for the revised model was 68.087. The revised model provided a better fit for data than the initial model.

To optimize the model, we removed paths not found to be significant and specified a revised model. Measures of model fit (χ^2^/df = 1.380, GFI = 0.938, AGFI = 0.859, CFI = 0.960, RMSEA = 0.070) indicated that the revised model had a satisfactory fit. Furthermore, given that the AIC of the revised model (68.087) was lower than that of the initial model, we concluded that that revised model provided better fit to data than the initial model. In the revised model, high self-esteem was observed to increase self-efficacy and to significantly influence QOL. In addition, low BASIS-32 was found to result in increased self-esteem and enhance QOL while high BASIS-32 increased the types of medications taken. Marital status (being married) was observed to positively impact self-efficacy and self-esteem (Figure 2). We investigated the effect of self-esteem on QOL of mental health patients by using the initial and revised models. With the initial model, the direct effect of self-esteem on QOL was 0.20. Its indirect effect, mediated by self-efficacy, was 0.55 + 0.17 = 0.72, making a total effect of 0.20 + 0.72 = 0.92. With the revised model, the direct effect of self-esteem on QOL was 0.20. Its indirect effect, mediated by self-efficacy, was 0.65 + 0.19 = 0.84, making a total effect of 0.20 + 0.84 = 1.04. This also indicated the goodness of fit of the revised model.

## Discussion

The importance of improving the QOL of disabled individuals to maintain and promote better lives of community-dwelling schizophrenic patients has been expounded upon in a wide variety of research. QOL is a subjective evaluation by individuals of their living situation that, accordingly, can only be defined using subjective measures. Skantze et al. [16] argued that subjective evaluations by patients of their own lives suggest that QOL depends more on the “inner experiences” than on “external experiences”. In other words, the “external world” represents “hard aspects” such as the environment in which patients live, while the inner environment represents thoughts and feelings. As such, Skantze et al. [17] argued that it is necessary, when evaluating QOL, to take into consideration patients’ physical, social, and cultural environments, that is, individuals’ living situations. In this study, we focused on this “inner world” and demonstrated that, within this inner world, self-efficacy and self-esteem, elements of self-concept, substantially impact the QOL of community-dwelling schizophrenic patients.

Although self-efficacy was identified as a factor influencing QOL, the mean total score for self-efficacy (5.39) among our study sample was substantially lower than the mean for healthy individuals reported by Sakano et al. [9]. Self-efficacy refers to individuals’ confidence in their ability to choose and carry out actions toward a certain goal or individuals’ confidence regarding their ability vis-à-vis expectations when carrying out such actions. Although there are many potential reasons for schizophrenic patients’ low self-efficacy scores, it is believed that the patients’ lack of life experiences resulting from the onset of disease, the paucity of experiences with success in everyday life, and the obstacles to participation in society resulting from the patient’s own sense of stigma serve to reduce patients’ sense of self-efficacy. It is thought that the “sense of shame” experienced by psychiatric patients up to this point accumulates with experiences of failure, resulting in a decline in sense of self-efficacy [18,19]. In this study as well, results of multiple regression analysis suggested that self-efficacy impacts various aspects of QOL. Furthermore, the concept of self-efficacy has been demonstrated to be an important mediating factor in the success of rehabilitation interventions as well as a factor influencing patients’ peace of mind [20]. From this and other results, it is believed that increasing patients’ sense of self-efficacy will lead to improved QOL, and that approaches such as cognitive behavioral therapy and social skills training that acknowledge psychosocial relations as well as approaches focusing on patients’ lives with the goal of improving actual behavioral patterns are important.

With regard to the relationship between self-esteem and QOL, results demonstrate that self-esteem is a necessary condition for high QOL. It is believed that, in the case of schizophrenic patients, high self-esteem arises from patients themselves constantly affirming their own thinking and ability to carry out daily tasks. Given the current emphasis placed on the QOL of schizophrenic patients and, keeping in mind that self-esteem is an important predictor of QOL, it is clear that providing assistance to help patients improve their self-esteem is extremely important [21,22]. As a disease that impacts the ego, schizophrenic patients face a grave threat to their sense of “self” as a part of their illness. For example, in delusions of control, the feeling that one is under the control of others substantially undermines self-esteem. Given that self-esteem is deeply tied with disease symptoms, it is necessary to develop treatment programs that take into consideration alleviation of symptoms as well as improvement of social skills.

It was also revealed that psychiatric symptoms are strongly related to the QOL of schizophrenic patients. In this study, we used BASIS-32 as a measure of psychiatric symptoms. Most QOL subscales were observed to be influenced by BASIS-32. Specifically, we found that skills related to daily tasks and establishment of one’s own role in daily life impact QOL. BASIS (daily living and role functioning), with a β = −0.537 (p < .0001), was observed to have an extremely strong impact on QOL (total). Award et al. [23] cited severity of psychiatric symptoms, side effects, and level of psychosocial functioning as the 3 most important determinants of schizophrenic patients’ QOL. In a pilot study, Lauer [24] reported observing a significant negative correlation between subjective QOL and acute psychiatric symptoms (Brief Psychiatric Rating Scale). Similarly, in our study, we observed a negative correlation between QOL and BASIS-32. Based on these results, we conclude that alleviation of psychiatric symptoms is an important factor for achieving higher QOL. Based on these findings, it is important that community-dwelling schizophrenic patients become more sensitive to psychiatric symptoms on a daily basis and that home care nurses and other health care providers work together with patients to develop strategies for dealing with symptoms when they occur.

In our study, the only socio-demographic factors found to be correlated with QOL were marital status, types of medications taken, and number of cigarettes smoked per day. Sex, age, educational level, and age at onset were not correlated with QOL. In the case of marital status (being married), it is believed that the presence of someone to talk to and consult with and the creation of a trusting relationship serve to increase QOL. In terms of types of medications taken, given that responses were provided by the schizophrenic patients themselves on a self-administered questionnaire, we were unable to confirm exact names of medications. As such, we were limited in identifying the types of medications taken. Despite this, a negative correlation was observed between the types of medication taken and QOL. Tobacco use and QOL (environment) were also found to be correlated, which makes sense. Lehman [25], studying schizophrenic patients staying in an overnight care facility in Los Angeles County, found that neither socio-demographic nor clinical characteristics of patients had a substantial impact on overall peace of mind. Lehman [26] reported that females, married individuals, and individuals with lower levels of education tended to be more satisfied with life than other residents. However, in a study involving interviews with schizophrenic patients visiting a community-based consultation service, Baker and Intagliata [27] reported no significant relationship between QOL and patient’s age or sex. As evidenced from the above, results of research vary to the point that it is difficult to identify a general trend.

By identifying factors affecting QOL and conducting structural analyses of those factors, we can determine the manner in which each factor impacts QOL. If a given factor can be improved, that relationship can be strengthened to achieve higher QOL. In the model developed in this study, the impact of items related to self-concept, self-efficacy, and self-esteem was large. Based on the multiple linear regression of QOL on BASIS-32 as a measure of psychiatric symptoms, it was found that QOL was substantially impacted by daily living and role functioning as well as social skills. It is conjectured that patients’ emotions associated with successful execution of social skills and feedback from others impact the patients’ level of self-concept. Focusing on self-esteem, which is a component of the self-concept, Baumgardner [28] argued that because individuals with low self-esteem have a negative image of themselves, while they seek positive feedback from others, what they need are people to acknowledge their skills. In this study as well, we found that marital status was correlated with QOL, and we constructed a structural model in which marital status (marriage) serves to increase self-efficacy and self-esteem. Accordingly, by proactively providing positive feedback regarding social skills, families of schizophrenic patients and care providers can help increase patients’ self-concept and, thereby, elevate their QOL. Self-concepts such as self-efficacy and self-esteem can be acquired through training. Cognitive behavioral interventions, of which the most well-known example is social skills training, represents an approach to supporting patients and family members that places psychosocial factors at the center. There are examples of research that have attempted to improve QOL by using such cognitive behavioral therapies [29,30]. From this, we see the importance of establishing places in the community where schizophrenic patients can assemble and interact, of offering assistance programs in the disability welfare service centers, of providing social skills training based on cognitive behavioral therapy, and of incorporating role playing into discussions among patients to nurture the belief that patients can accomplish things on their own and thereby improve patients’ self-concept and QOL.

### Study limitations

The first limitation of the present study is that it analyzes the QOL of schizophrenic patients living in the community at a single point in time. As such, any speculation regarding causal relationships must be made with caution. Initially, we hypothesized that marital status (being married) directly enhances QOL and that patient’s age directly impacts self-esteem and self-efficacy. However, it could be the case that being married expands one’s sphere of social relationships and, as a result, increases one’s self-esteem and self-efficacy. By carefully examining each circumstance in this manner, we can explore the mechanisms by which the factor contributes to QOL. It is necessary to conduct a follow-up survey related to the patients’ QOL in the future. The second limitation is related to the fact that data for this research were collected via a questionnaire survey. In questionnaire surveys, respondents’ choices for responses are limited. Structured interviews would enable researchers to hear and analyze the subjects’ true voices. While such interviews are labor-intensive, they would allow researchers to think about QOL based on a broader range of factors including family, psychiatric treatment, the future, and employment. Based on the results of this study, we plan to continue analyzing factors contributing to QOL.

## Conclusions

The factors observed to influence QOL included self-efficacy and self-esteem, 2 components of self-concept, as well as a complex interweaving of factors such as psychiatric symptoms, type of medication, patient’s age, and marital status. Self-efficacy was found to have an especially strong and direct impact on QOL. As such, it is important to provide more positive feedback to patients, provide social skills training based on cognitive behavioral therapy, and engage patients in role playing to improve self-efficacy and self-concept.

## Competing interests

The authors declare that they have no competing interests.

## Authors’ contributions

HN, NW, and EM conceptualized the rationale and design of the study. EM advised HN about data management for SPSS. HN, NW, and EM conducted statistical analyses and drafted the manuscript. All authors read and approved the final manuscript.
